# Supplementary material for: Collective polarization dynamics in bacterial colonies signify the occurrence of distinct subpopulations
Source: PLoS Biol. 2023 Jan 18;21(1):e3001960. doi: 10.1371/journal.pbio.3001960 (PMC9847958; doi:10.1371/journal.pbio.3001960)
Supplement: S1 Text — (PDF) [file pbio.3001960.s001.pdf]

Supporting information for

Collective polarization dynamics in bacterial colonies signify the occurrence of distinct subpopulations

Marc Hennes, Niklas Bender, Tom Cronenberg, Anton Welker, Berenike Maier

Institute for Biological Physics, and Center for Molecular Medicine Cologne,

University of Cologne, Cologne, Germany

Supporting Figures

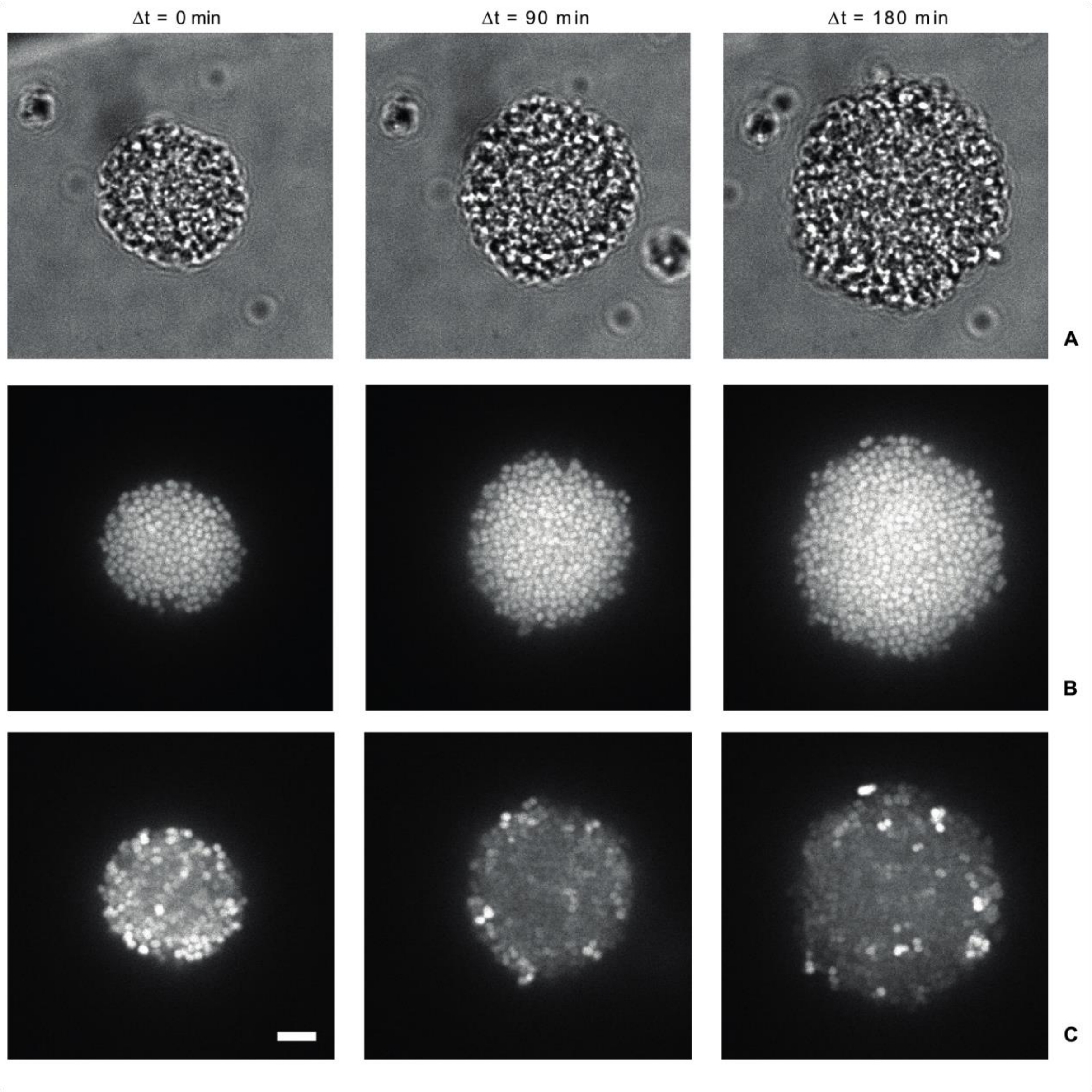

Figure i. Comparison between TMRM and sfGFP fluorescence in colonies. Strain $wt\ green$ (NG194) in flow chamber. A) Brightfield images of colonies imaged at the indicated time points. B) sfGFP fluorescence. C) TMRM fluorescence. Scale bar: 5 µm.

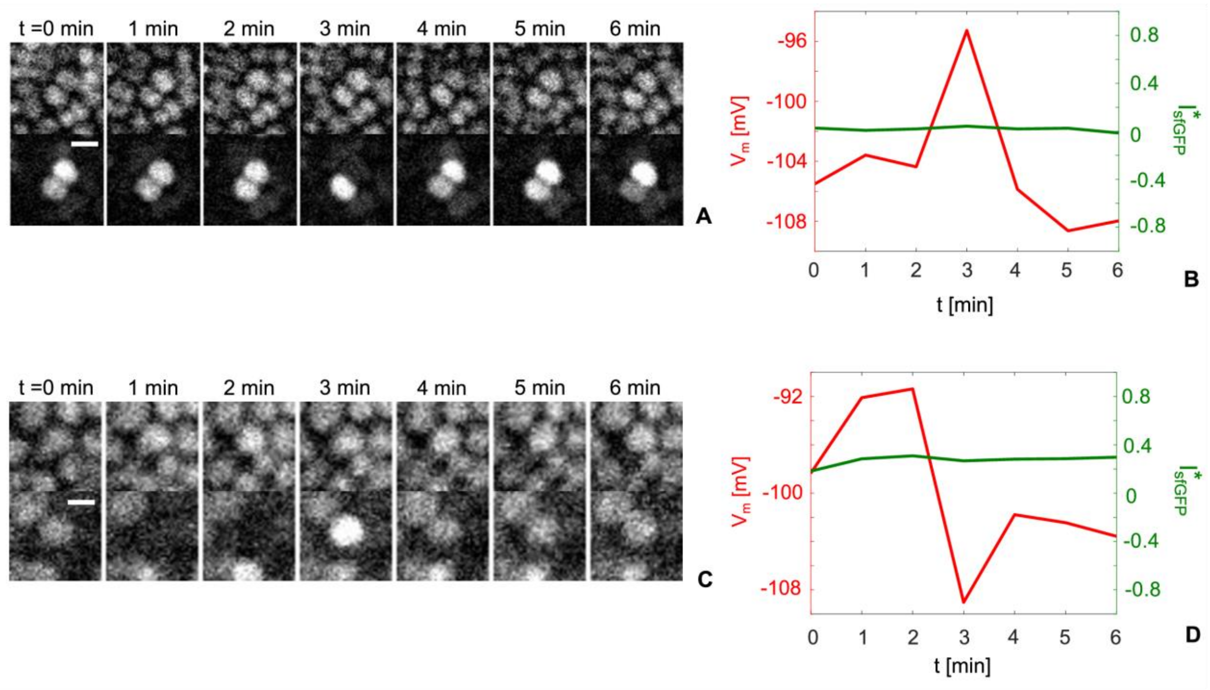

Figure ii. Transient depolarization and hyperpolarization events of single cells occur on the time scale of minutes and are uncorrelated between neighbours. Strain $wt\ green$, NG194, flow chamber. A) sfGFP (top) and TMRM (bottom) signal time lapse during a transient depolarization event. Δt = 1 min. B) Membrane potential (left axis) and normalized sfGFP-signal (right axis) $I^*_{sfGFP} = (I_{sfGFP} - < I_{sfGFP} >_{cells}) \, / < I_{sfGFP} >_{cells}$ for the time lapse of A). C) sfGFP (top) and TMRM (bottom) signal time lapse during a transient hyperpolarization event. Δt = 1 min. D) Membrane potential (left axis) and normalized sfGFP-signal (right axis) for the time lapse of C). (See S1 Data for raw values) Scale bar: 1 μm

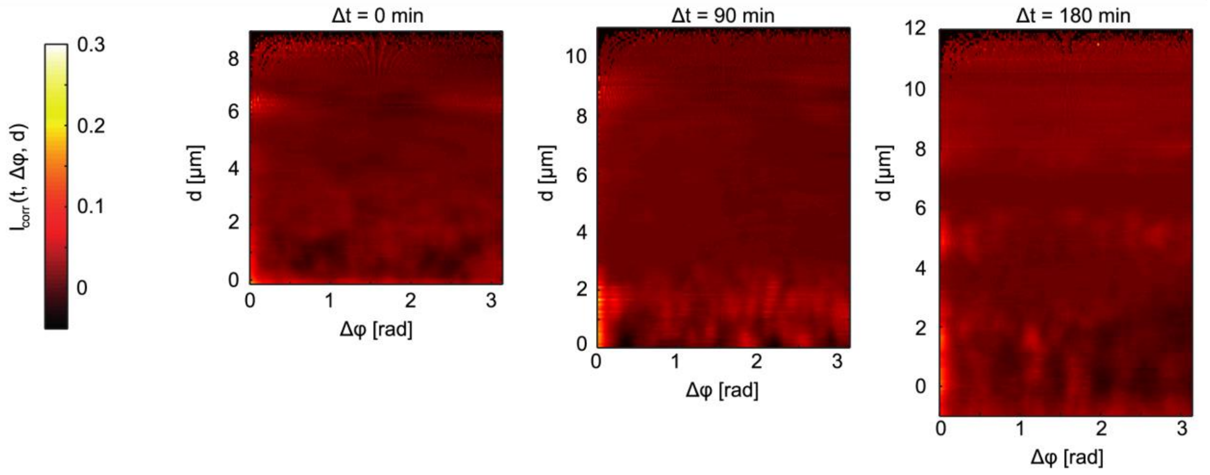

Figure iii. Further example of angular correlation of the intensity fluctuations within the colony prior to the transition

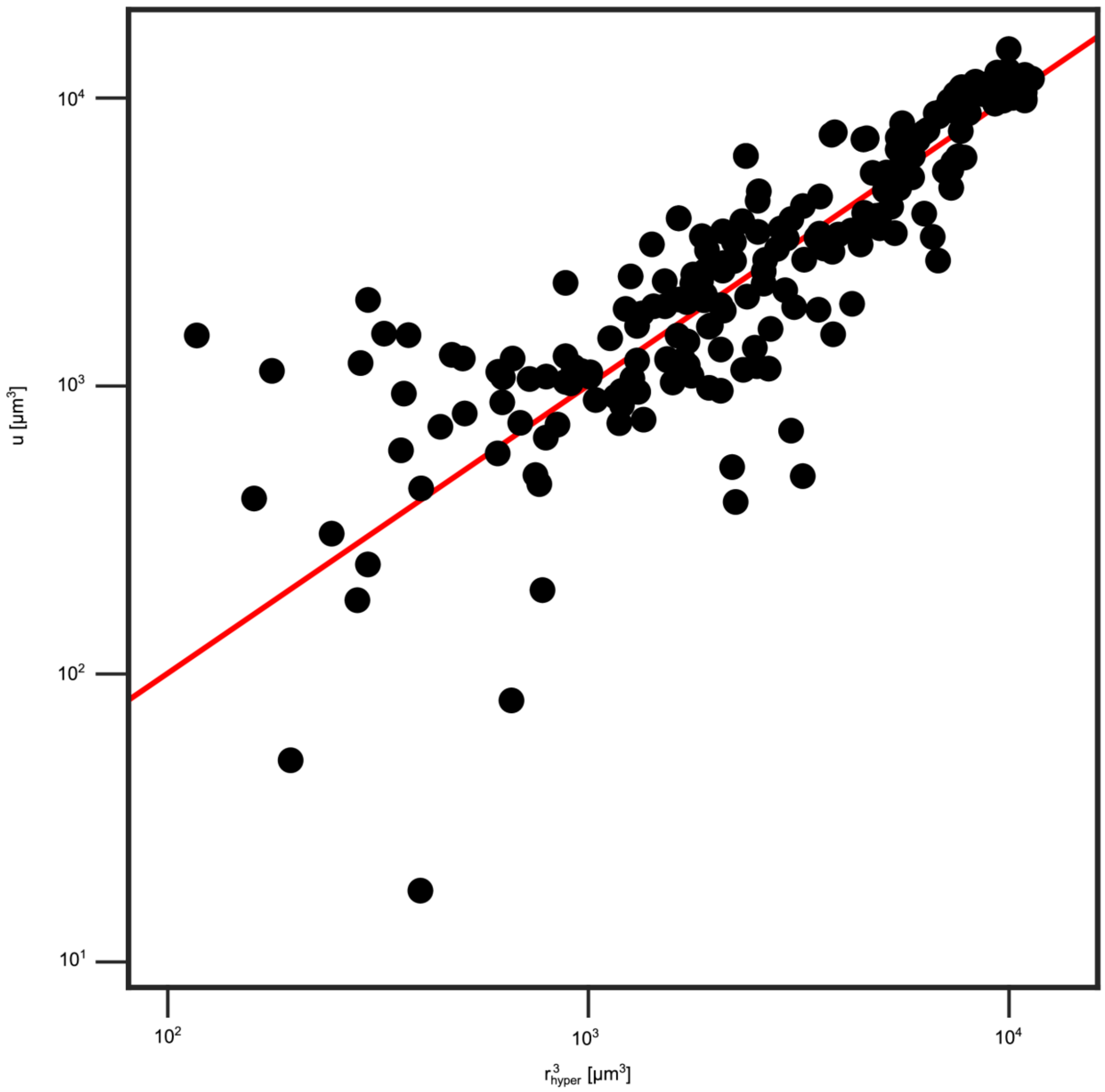

Figure iv. Relation between the cubed hyperpolarized shell radius r_{hyper}^{3} and the colony radius factor $u \equiv R^2\left(R - \frac{3\dot{R}}{\lambda_0}\right)$. Strain *wt green*, NG194, flow chamber. Each point represents one measurement of both variables (15 colonies evaluated). Red line: fit to power function $y = x^b$ ($b = 1.006 \pm 0.004, R^2 = 0.86$). (See S1 Data for raw values)

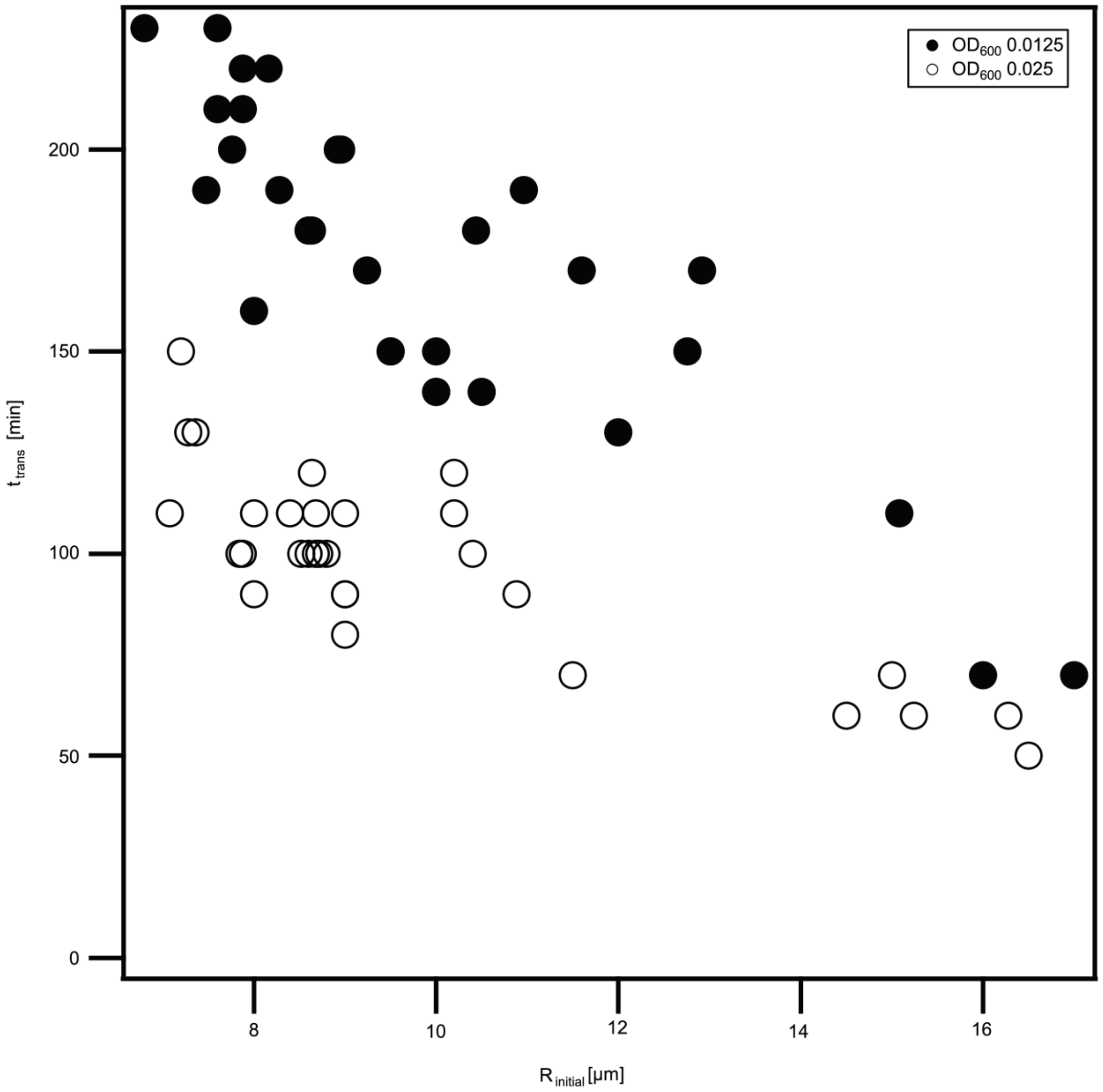

Figure v. The time point of transition to collective behaviour depends on initial colony size and cellular concentration. Strain $wt\ green$, NG194, static culture. The inoculation densities were OD_{600} 0.0125 (filled circles) and OD_{600} 0.025 (open circles), respectively. (See S1 Data for raw values)

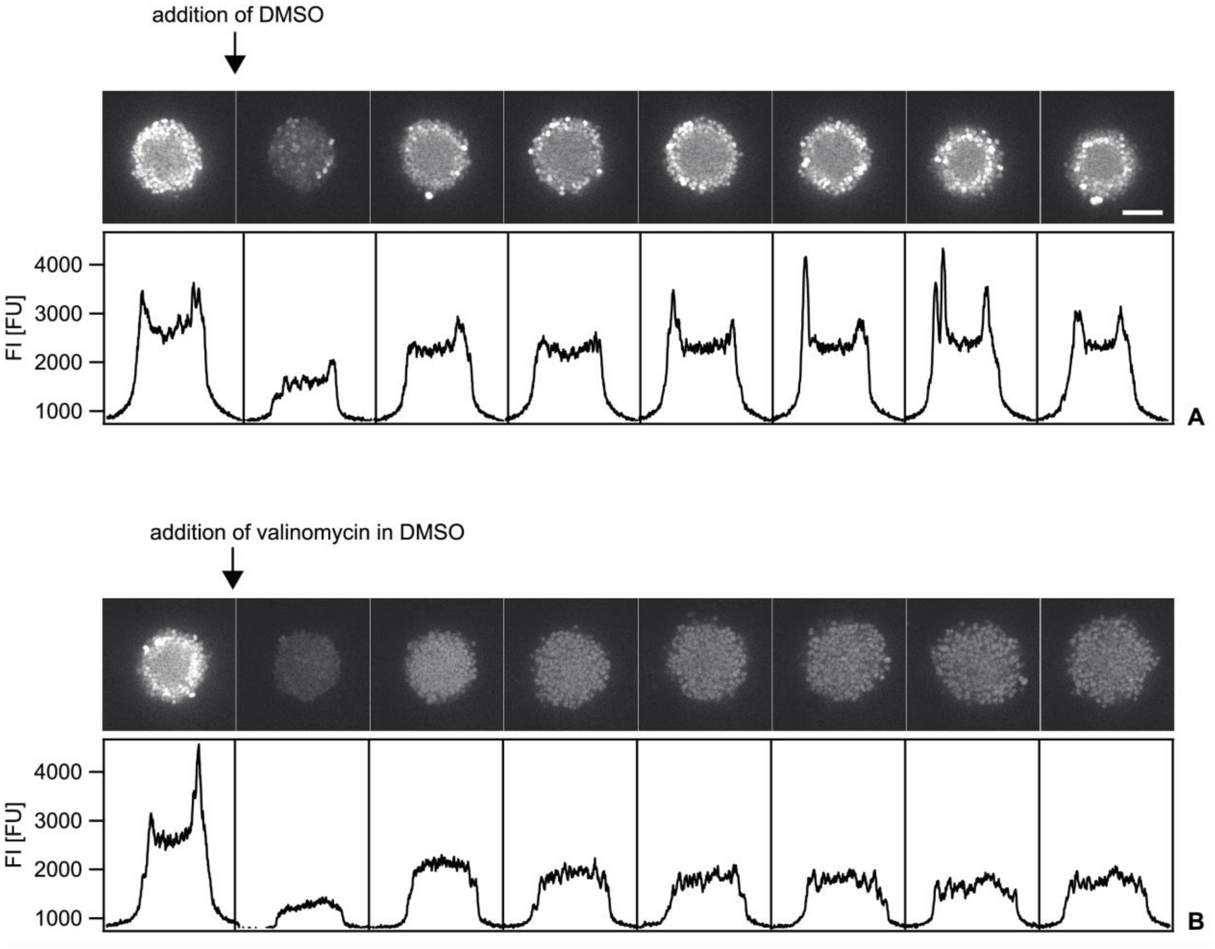

Figure vi. Addition of the K$^+$ selective ionophore homogeneously depletes the membrane potential. Strain wt^* (NG150), static culture. After the shell of hyperpolarized cells had travelled to the edge of the colony, valinomycin was added. A) Control. DMSO was added at the indicated time point. Immediately after addition of DMSO, cells depolarized transiently. Within 5 min, the shell had re-formed and the membrane potential pattern was restored. B) Valinomycin in DMSO was added to a final concentration of 3 µM. After the initial depletion, the membrane potential was only partially restored and no hyperpolarization was evident. Colonies started to disassemble. (See S1 Data for raw values) Δt = 5 min. Scale bar: 10 µm.

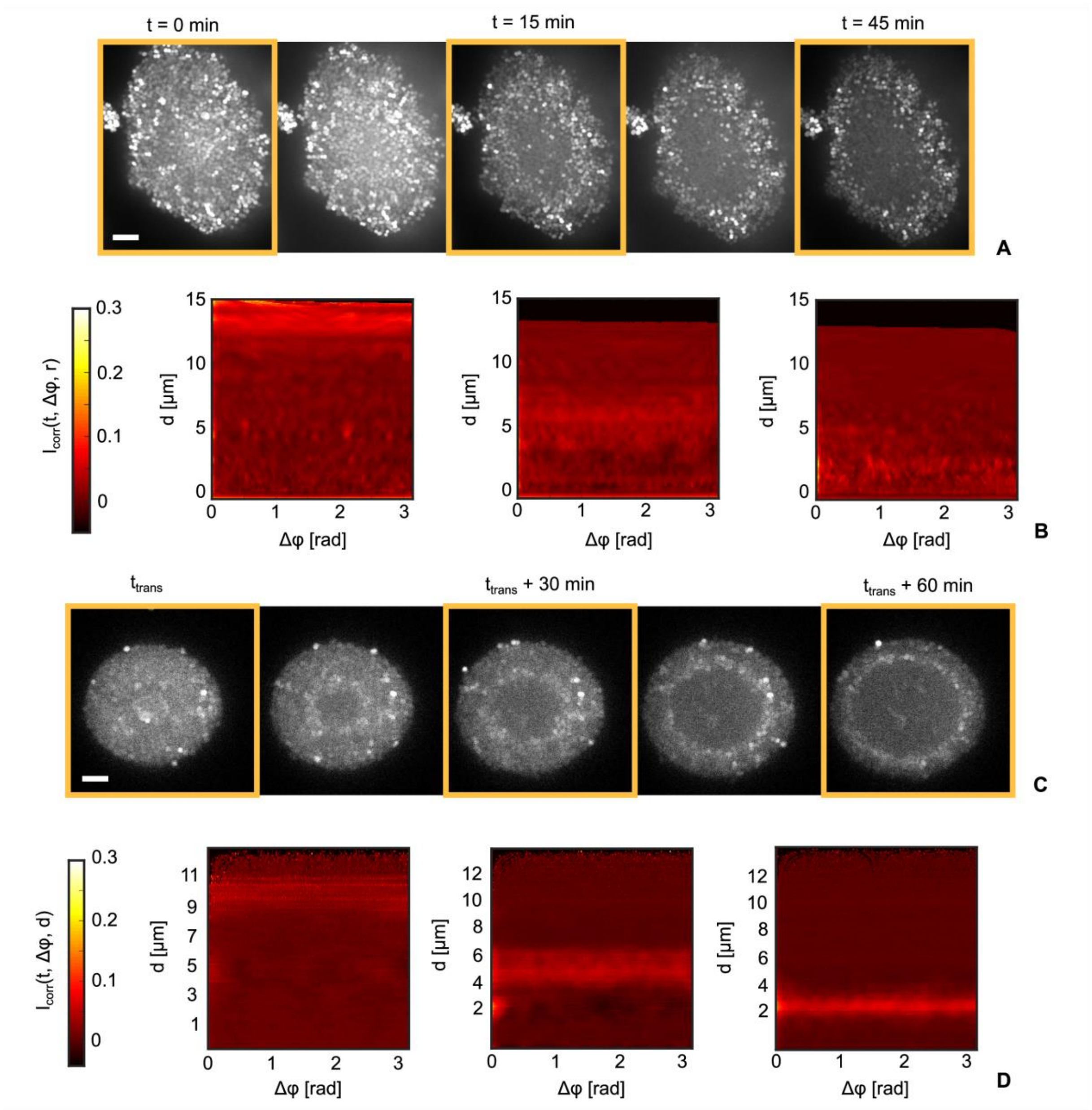

Figure vii. Membrane potential dynamics for a retraction-deficient and retraction-reduced strain. Flow chamber. A) Typical time lapse of TMRM fluorescence of the T4P retraction deficient strain *ΔpilT* (NG231). Scale bar: 5 μm. B) Angular correlation of the intensity fluctuations as a function of radial position for strain *ΔpilT* at time points t=0, 15 min, 45 min (t=0 min: start of colony centre depolarization). Images correspond to timeframes in A) marked by orange boxes. C) Typical time lapse of TMRM fluorescence of strain $pilT_{WB2}$ (NG176). Scale bar: 5 μm. D) Angular correlation of the intensity fluctuations as a function of radial position for strain $pilT_{WB2}$. Images correspond to timeframes in C) marked by orange boxes. (See S1 Data for raw values)

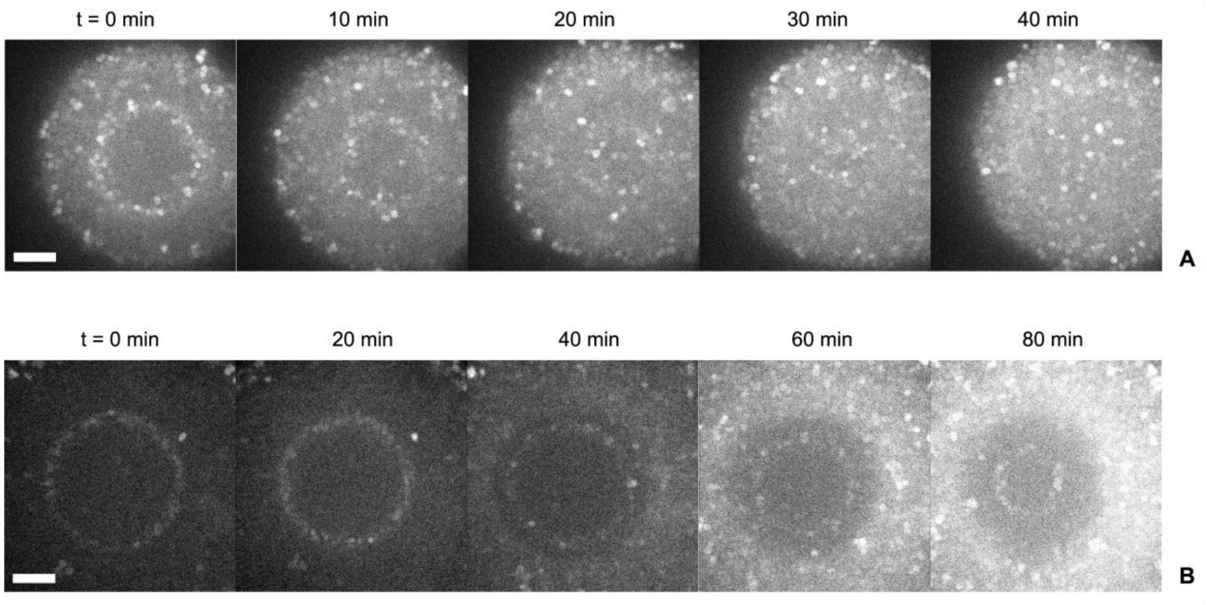

Figure viii. Dynamics of membrane potential after azithromycin treatment. Strain wt^* (NG150), flow chamber. Colonies were treated for 30 minutes with azithromycin at MICx100, $0.64\ \mu g/ml$ (A, B : two different colonies), and exhibit within 1 to 2 hours after treatment a collective hyperpolarization and shell reversal. Scale bar: 5 µm.

Supporting Table

Strain	Genotype	Source
wt^{*} (NG150)	*G4::aac*	[1]
$wt\ green$ (NG194)	*lctp:PpilE*<br>*sfgfp speR:aspC*<br>*G4::aac*	[2]
$\Delta pilT green$ (NG231)	*pilT::m-Tn3cm*<br>*lctp:PpilE sfgfp speR:aspC*<br>*G4::aac*	[3]
$pilT_{WB2}$ (NG176)	*iga::*P_{pilE} $pilT_{WB}$ *ermC*<br>*G4::aac*	[4]

Table i. Strains used in this study

Supporting References

1. Zollner R, Oldewurtel ER, Kouzel N, Maier B. Phase and antigenic variation govern competition dynamics through positioning in bacterial colonies. Sci Rep. 2017;7(1):12151. Epub 2017/09/25. doi: 10.1038/s41598-017-12472-7. PubMed PMID: 28939833; PubMed Central PMCID: PMCPMC5610331.

2. Welker A, Hennes M, Bender N, Cronenberg T, Schneider G, Maier B. Spatiotemporal dynamics of growth and death within spherical bacterial colonies. Biophys J. 2021;120(16):3418-28. doi: 10.1016/j.bpj.2021.06.022. PubMed PMID: WOS:000686346200020.

3. Hennes M, Cronenberg T, Maier B. Caging dynamics in bacterial colonies. Phys Rev Res. 2022;4(1). doi: ARTN 01318710.1103/PhysRevResearch.4.013187. PubMed PMID: WOS:000768406800002.

4. Welker A, Cronenberg T, Zollner R, Meel C, Siewering K, Bender N, et al. Molecular Motors Govern Liquidlike Ordering and Fusion Dynamics of Bacterial Colonies. Phys Rev Lett. 2018;121(11):118102. Epub 2018/09/29. doi: 10.1103/PhysRevLett.121.118102. PubMed PMID: 30265121.
