# Supplementary material for: Collective polarization dynamics in bacterial colonies signify the occurrence of distinct subpopulations
Source: PLoS Biol. 2023 Jan 18;21(1):e3001960. doi: 10.1371/journal.pbio.3001960 (PMC9847958; doi:10.1371/journal.pbio.3001960)
Supplement: Attachment — Submitted filename: Response.pdf [file pbio.3001960.s006.pdf]

Reviewer #1: The manuscript by Hennes et al. investigates the role of membrane potential during aggregation of individual cells of N. gonorrhoeae. The authors identified a striking behavior or hyperpolarization in colonies that correlated with a change in growth behavior of the colony. The hyperpolarization was simulated which suggested oxygen depletion as a critical initation event for the hyperpolarization which was supported by experiments. Finally, they show a heterogeneous response to cell survival depending on the state of polarization in the colony. Overall, I believe the authors found a very interesting behavior which was highly reproducible with important physiological consequences. The paper was well written and clearly presented. I have only a few issues to contribute to this excellent manuscript.

We thank the reviewer for her or his positive feedback.

1. The identification of K+ as the responsible ion. I'm not sure the authors can definitively say that K+ is ion dictating the hyperpolarization. When using valinomycin, it acts as a voltage clamp by enabling an ion specific flow. So it will set the voltage of all the cells to whatever is the Nerstian concentration difference between inside and outside the cells. The data helps confirm that it is indeed voltage hyperpolarization, but does not necessarily suggest K+. For example, changes in Na+ could be occurring, but those changes would just be offset by a flow in K+ resulting in the clamped cell.

We agree. We did not mean to state that K+ ions dictate hyperpolarization. We have reformulated the sentence that might imply such a statement.

Second, the experiments with the potassium indicator were confusing. In many conventional cells (neurons, cardiomyocytes, E. coli), the potassium concentration inside the cell is high relative to the outside, such that it sets up a negative polarization. If one increases the cytoplasmic concentration of calcium, that would result in an increase in voltage (hyperpolarization). What I don't know is the concentration of K+ inside N. gonorrhoeae. If it is much lower than the medium concentration, it is possible that increased K+ could depolarize. A second alternative could be that the pH inside the cells is changing after reaching a critical O2 level. The crown-ethers are particulary sensitive to pH. If the authors want to make a stronger claim about the importance of K+, they should do the genetics (as in the Suel Nature paper), or use a fluorescent pH indicator to show that pH is not responsible for the APG-2 fluorescence changes. However, I believe the authors could change the language and cage their findings a bit without hurting the impact of the manuscript, too.

We don't fully understand the reasoning of this paragraph. In particular, we are confused about the term "hyperpolarization". The overall electrical membrane potential of the gonococci is negative, around - 90 mV. To our understanding, hyperpolarization means that the electrical potential becomes

even more negative, e.g. -100 mV. This definition of hyperpolarization was also used for example in Bruni & Kralj (doi.org/10.7554/eLife.58706) or in Stratford et al (doi/full/10.1073/pnas.1901788116). If we observe an influx of K^+ ions, then the net negative charge density within the cell will decrease and, as a consequence, the membrane potential will be less negative, e.g. -80 mV. We call this depolarization.

An influx of K^+ ions would increase the entropic component of the proton motive force, but we don't discuss pmf here. Therefore, we think that it is valid to say that "This finding suggests that depolarization is at least partially caused by and influx of K^+ ions." We fully agree that additionally H^+ concentrations, Na^+ concentrations, or Ca^{2+} concentrations may change. Unfortunately, we don't have a laser that would enable us to use the ratiometric H^+ sensors. Probing extra- and intracellular concentrations of the other ionic species would be very expensive and, therefore, we decided not to proceed with the experiments, since this aspect is not the main focus of the work. We have added a sentence stating "We cannot exclude, however, that other ionic species are involved in the formation of the spatial polarization pattern."

2. Small issue: After seceral reads, the data such as in figure 3c looked like time traces to me. I thought that the colonies repeatedly went high and then low as the x-axis is labeled in minutes. Can you separate the traces to make it clear that you are looking at a spatial profile for 1 time point, not a continuous time trace?

We thank the reviewer for pointing this out. We have adjusted the figure.

3. I would bring the data in S6 (oxygen depletion) to the main figure. The appearance of the shell at the outer edge is visually striking and I think it's worth elevating it to give it more impact.

We agree with the reviewer and have moved Fig. S6 to Fig. 4f.

4. For the claims of no changes in extracellular ions, is there data that could be shown in the supplement?

As explained in the response to the first point, we have removed this sentence and instead write "We cannot exclude, however, that other ionic species are involved in the formation of the spatial polarization pattern."

Reviewer #2: In this manuscript Hennes et al. explore the spatial dynamics of growth and membrane hyperpolarization/depolarization that occur in aggregated colonies of Neisseria gonorrhoeae (Nh). Using confocal microscopy, cellular resolution analysis, and a variety of fluorescent dyes they demonstrates that membrane polarization patterns are disordered in aggregates of Nh right after they have assembled via T4P activity. Shortly thereafter, they observe a spherical wave of hyperpolarization that proceeds from the colony center and travels toward the outer periphery, stopping several cell lengths from cell-liquid interface. Behind this wave, cells depolarize slightly and cell growth arrests. They show that the depolarization region behind the traveling shell corresponds to influx of potassium into the cells, and they use a reaction-diffusion model to support the interpretation that this polarization wave occurs together with the decrease in local oxygen concentration threshold required for cell growth and division. Supplementation with an alternative electron acceptor eliminates the formation of the polarization wave, at least on the time scale of these experiments. The growth-arrested region of the cell groups in their interior also corresponds to local tolerance to kanamycin.

Overall I found this to be a very strong paper with good technical execution throughout, and explained very clearly in the text. It is VERY much appreciated that the authors ****did not**** *succumb to the temptation to characterize this pattern of colony activity as a kind of analogue to neural activity, or embryo development, as other groups have recently done for Bacillus subtilis - in my opinion, with no real justification on logical or empirical grounds and at significant expense to the clarity of the biofilm research community.*

I have a few minor comments about a few points in the text, and a broader comment about some of the phrasing in the introduction and discussion that the authors may wish to consider. Beyond this I think this paper will be well received in the community and is a good fit to the general readership of this journal.

We thank the reviewer for her or his positive feedback.

1) The figure panels are not labelled very clearly - there are little letters in the bottom right of the multiple panel set to which the letters correspond. At first I did not see them, which made the figures a bit harder to read than they need to be. I would switch the letters to larger bolded versions in the top-left of each corresponding panel.

To improve clarity and readability, we have enlarged and boldened the figure panel labels everywhere.

2) Lines 187-189: it is noted that extracellular concentrations of K+ and N1+ were measured, as were intracellular concentrations of Ca2+ . . . but there is no reference to main text or SI figures in this sentence.

We have removed this sentence and instead write "We cannot exclude, however, that other ionic species are involved in the formation of the spatial polarization pattern."

3) Lines 198-200: the paper states here that local oxygen concentration is a primary factor for the chemistry and physiology of a microenvironment. This is certainly true in many cases, but not universally true, e.g. in anaerobic environments and for bacteria that are not obligate aerobes like Nh (though it can use nitrite as an alternative e- acceptor). I would just change the wording here slightly so as not to overgeneralize.

We have toned the statement down to say that oxygenation is an important factor.

4) Lines 307-309: Can it be said for certain that the depolarization increases tolerance against kanamycin, or could it just be growth arrest, for which depolarization is a physiological indicator?

A recent publication by Bruni & Kralj (doi.org/10.7554/eLife.58706) shows that the killing activity of aminoglycosides is related to membrane potential. Therefore, we think that most likely reduction of membrane potential reduces the killing activity of kanamycin. We agree, however, that we have not formally shown that growth arrest is not involved. Therefore, we removed the half-sentence concerning polarization-mediated tolerance from the Results part and added a few sentences in the Discussion

5) Line 336: Suggest changing to "Depending on the oxygen consumption rates and the exogenous oxygen supply concentration …" To this point, there is a substantial literature from the biofilm simulation field on the reaction-diffusion processes that govern the depth of actively growing layers in biofilms. These may be good to cite here. See in particular: PMID: 10099266

We have changed the sentence as suggested by the reviewer and added a few sentences concerning reaction-diffusion processes.

6) There is some language in the introduction and conclusion that makes it a bit ambiguous as to whether the authors think that groups of Nh cells are generating these polarization/depolarization "on purpose"; that is, whether they evolved to do this, or whether it is just a biophysical consequence of growing in a group with a limiting solute diffusing from the exterior and being depleted in the interior. The simplest explanation in my view would be the latter view; the cells do not have a regulatory switch from disordered to collective behavior, rather this transition occurs as a consistent outcome of

the cell group geometry, cell nutrient/oxygen consumption, and nutrient/oxygen supply conditions. The majority of the language in the main text seems to imply this view, but there is text in the introduction and discussion that sometimes seems to imply that this is all part of an active, regulated differentiation process, rather than a byproduct of cell group growth. This was the only real point where the clarity of the test faltered somewhat to my reading, so I would advise taking a look out for this in a revision.

We thank the reviewer for pointing this out. We did not mean to imply that there is a genetic switch that causes collective polarization. To avoid confusion, we have changed the word "switch" to "transition" throughout the manuscript and, additionally, changed the wording in the introduction.

Reviewer #3: This manuscript describes a previously unknown collective behaviour in developing colonies of the bacterium Neisseria gonorrorhea: a transition from uncorrelated, random polarization fluctuations of individual cells, to directional waves of hyperpolarization. The authors show convincingly that this phenomenon is triggered by oxygen depletion within the growing colony and is connected with growth arrest. This discovery is intriguing and potentially important since it may have wider relevance for biofilm development. The manuscript is well written and the findings are convincingly supported by data. I have a number of comments:

We thank the reviewer for her or his positive feedback.

1. The manuscript's conclusions rely heavily on the use of the Nernstian dye TMRM to measure membrane potential. The relationship between the readout of Nernstian dyes and membrane potential is not always simple, so that careful checking and calibration is needed (see, e.g. Mancini et al, (2020) Biophysical Journal 118, 4-14). In this manuscript the conclusions are mainly qualitative but it would still be reassuring to include some discussion on the validity of the dye for quantiative measurements.

We have added discussion of this topic in the Methods section. Furthermore, we now refer to potential pitfalls of membrane potential quantification in the main text. As pointed out by the reviewer, the conclusions of our work do not rely on the quantitative analysis of the membrane potential.

2. Prior to the hyperpolarization wave, it is asserted that the membrane potential shows no spatial correlation (eg line 102 "a randomly distributed subpopulation maintaining strong polarization"). However, Figure 2b does seem to show some spatial structuring of the fluorescence, with

brighter cells preferentially located at specific radii. It would be good to comment on this.

We have added a sentence to the text explaining that the polarization of diplococci is apparently correlated. Additionally, we cannot exclude that a random fluctuation sometimes causes a small correlation, as is the case for Fig. 2b. However, we show multiple examples (including Fig. S3) with very little correlation compared to the signal we find during transient hyperpolarization.

3. Is this random polarization and depolarisation that is seen at early times also seen in planktonic cells? I would have liked to see some discussion of whether it is a feature of early colony formation or a remnant of planktonic behavior.

We agree that it would be very interesting to investigate the polarization dynamics of individual gonococci. However, this topic is beyond the scope of this work. At this point, we don't want to speculate about the potential results of future experiments.

4. Lines 143-6 "We determined the radii of individual colonies as a function of time (start radius 0 and growth rate 0) and normalized it by the radial growth function of the first 2 hours…" for me this needed more explanation. An additional sentence should be added to explain where the expression for the radial growth function in the first 2 hours comes from.

We extended the explanation of the radial growth function.

5. Lines 157-8 It is stated that the wave of hyperpolarisation precedes growth arrest. But the evidence that hyperpolarization comes first then growth arrest (not the other way around) could be made clearer. Perhaps this point could be strengthened?

We thank the reviewer for pointing this out. We have changed the word "precedes" to "coincides" since we don't know whether hyperpolarization or growth arrest comes first.

6. Lines 299-301 It is intriguing that treatment with kanamycin apparently reverses the direction of the wave of hyperpolarization. The authors suggest in the discussion that this could happen because kanamycin prevents growth and hence depletion of oxygen. Perhaps this hypothesis could be tested using the already developed simulation model for oxygen dynamics.

As suggested by the reviewer, we ran the simulations. If the growth rate in the whole colony is set to zero, we observe that the propagation of the iso-concentration circle in the simulations stops (in less than one minute), as

oxygen uptake is locally balanced/equilibrated by oxygen diffusion. Additionally, if the oxygen uptake rate is globally lowered, the direction of the iso-concentration propagation circle can be reversed, as diffusion takes over oxygen consumption. However, a match between reversed propagation velocities/characteristics in experiments and simulations would require knowledge about the rate of change and final levels of oxygen consumption rates under kanamycin/azithromycin treatment. We would like to address and perform these measurements in future work and have opted to not add the preliminary data on simulated propagation reversal.
